# Two new species of *Satsuma* A. Adams, 1868 from Taiwan (Pulmonata, Camaenidae)

**DOI:** 10.3897/zookeys.795.28958

**Published:** 2018-11-08

**Authors:** Chung-Chi Hwang, Shu-Ping Wu

**Affiliations:** 1 Department of Life Sciences, National University of Kaohsiung, No.700, Kaohsiung University Road, Nan-Tzu District, Kaohsiung 81148, Taiwan National University of Kaohsiung Kaohsiung Taiwan; 2 Department of Earth and Life Sciences, University of Taipei, No.1, Ai-Guo West Road, Taipei 10048, Taiwan University of Taipei Taipei Taiwan

**Keywords:** anatomy, Gastropoda, land snail, sinistral, Stylommatophora, taxonomy

## Abstract

Two new sinistral species of the genus *Satsuma* A. Adams, 1868, *Satsumasquamigera***sp. n.** and *Satsumaadiriensis***sp. n.**, from southern Taiwan are described. *Satsumasquamigera***sp. n.** is characterized by a microsculpture comprising coarse, irregularly-spaced ridges and dense, easily-dislodged triangular scales on its sinistral shell, an angulated periphery, and partly-opened umbilicus. This species inhabits secondary forests in lowland hills. *Satsumaadiriensis***sp. n.** is characterized by a thin, fragile smooth shell with microsculpture of coarse, loose ridges, a rounded periphery, completely-opened umbilicus, and elongated penial verge formed by two main pilasters. This new species was collected in a mountainous, mid-elevation, broad-leafed forest.

## Introduction

The family Camaenidae, which includes the confamilial Bradybaeninae, is widely distributed in Asia and Australasia (Wade et al. 2007). Recent studies have elucidated the systematics of this family by means of molecular tools (e.g., Wade et al. 2007, Hoso et al. 2010, Criscione and Köhler 2014), however significant gaps persist in the documentation of local faunas, such as in the genus *Satsuma* A. Adams, 1868. This genus is distributed in East Asia (Schileyko 2004), containing more than 100 species inhabiting Japan, China, Philippines, and Taiwan (Minato 1988, Wang et al. 2014, Adams and Reeve 1850). Some Vietnamese species, currently assigned to other genera, are likely part of genus *Satsuma* as well (Schileyko 2011). Species of *Satsuma* are characterized by conical, brownish shells varying in shape, size, color, chirality and banding (Schileyko 2004). The reproductive system of this genus features an epiphallic flagellum and a penial caecum, while dart sac, accessory sac, and mucous glands are absent (Kuroda and Habe 1949, Schileyko 2004).

To date, 46 species have been described from Taiwan; most of them are endemic to Taiwan and narrowly distributed (Hsieh et al. 2013, Wu and Tsai 2014, 2015, 2016, Wu and Wu 2017a, 2017b, Hwang et al. 2017). Previous studies have suggested that there are potentially undescribed species in Taiwan, especially in mountainous areas (Wu et al. 2007, 2008, Hwang et al. 2017). In this study, we describe two new Taiwanese species from mountainous areas of lowland and mid-elevation, based on shell morphology and genital anatomy.

## Materials and methods

**Figure 1.**
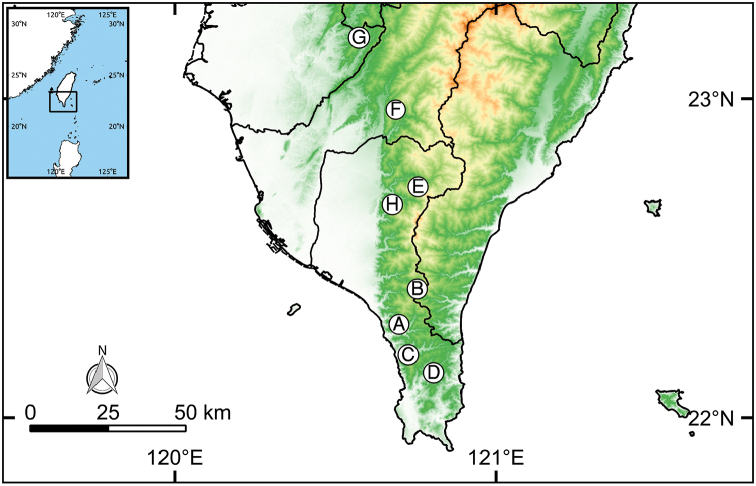
Distribution map of two *Satsuma* species in southern Taiwan. **A–D***S.squamigera* sp. n.: **A** Ka-yo-fong waterfall, Shih-tze, Pingtung (type locality) **B** Da-han- shan forest road, Pingtung **C** Mt. Bei-li-long, Pingtung **D** Mu-dang, Pingtung; **E–H***S.adiriensis* sp. n.: **E** A-li, Wu-tai, Pingtung (type locality) **F** Shan-ping, Liu-guei, Kaohsiung **G** Mt. Fan-bao-jian, Nan-xi, Tainan **H** Ma-jia, Pingtung.

Specimens of the new species were collected in southern Taiwan (Figure 1). Live adults were drowned in water for 12 hours, then boiled briefly in hot water at 95 °C. Whole snails were fixed and preserved in 95% ethanol. Immediately before dissection, the snails’ tissues were softened with warm water, and the body was removed from the shell. Empty shells were then cleaned, oven-dried, and stored at room temperature. Reproductive systems were dissected under a stereomicroscope (Leica MZ7.5). Drawings were made using a camera lucida attachment. We used the methods described by Kerney and Cameron (1979) to measure shell characteristics to 0.1 mm and to count the number of whorls to 0.25 whorls. Measurements of genitalia were obtained from the digital images using ImageJ 1.48k (Schneider et al. 2012). We followed Gómez’s (2001) terminology in describing the reproductive system. The WGS84 coordinates of localities were recorded. A distribution map was created using the open-source software Quantum GIS 2.18.1 (QGIS Development Team 2016) with topographic databases ASTER GDEM V2 released by NASA and METI (downloadable from https://asterweb.jpl.nasa.gov) and GADM 2.8 released by Global Administrative Areas (downloadable from http://gadm.org/). The type specimens have been deposited in the National Museum of Natural Science, Taichung, Taiwan (NMNS).

**Abbreviations**

**NMNS** National Museum of Natural Science, Taichung, Taiwan.

Genitalia:

**ag** albumen gland;

**at** atrium;

**bc** bursa copulatrix;

**ep** epiphallus;

**fl** flagellum;

**fod** free oviduct;

**p** penis;

**pc** penial caecum;

**pd** pedunculus of the bursa copulatrix;

**rm** retractor muscle;

**sod** spermoviduct;

**v** verge;

**va** vagina;

**vd** vas deferens.

Shell measurements:

**AH** aperture height;

**AW** aperture width;

**SH** shell height;

**SW** shell width;

**W#** number of whorls

## Systematics

### Family Camaenidae Pilsbry, 1895

#### *Satsuma* A. Adams, 1868

**Type species.***Helixjaponica* Pfeiffer, 1847, by subsequent designation (Kuroda and Habe 1949: 54)

##### Satsuma squamigera sp. n.

Taxon classificationAnimaliaStylommatophoraCamaenidae

http://zoobank.org/DAAF8145-928F-4755-8132-B1FDF89A4509

Figures 1 , 2 , 3

###### Type material.

**Holotype**NMNS-7944-001, dry shell and dissected soft part in ethanol, coll. C. C. Hwang, 19 May 2016, collected from type locality; paratype NMNS- 7944-002, 1 specimen: dry shell and dissected soft part in ethanol, coll. S.P. Wu, 24 Jul 2014, collected from type locality; paratypes NMNS-7944-003, 5 specimens: 5 dry shells and 1 dissected soft part in ethanol, coll. S.T. Yang, 11 Feb. 2011, collected from type locality, paratypes NMNS-7944-004, 4 specimens: dry shells, coll. C. C. Hwang, 19 Aug 2014, collected from type locality, paratypes UTM2018001-5, 5 specimens: dry shells, coll. S. P. Wu, 11 May 2012, collected from type locality.

###### Type locality.

Taiwan: Pingtung County, Shih-tze, Ka-yo-fong waterfall (also named Nei-shih waterfall), 22°17.55'N; 120°41.88'E, alt. 170 m, secondary lowland broad-leafed forest (Figure 1A).

###### Diagnosis.

Shell sinistral with coarse and irregularly ridged and fine striations; surfaces with dense, fine, erected, triangular scales falling off easily; periphery angulated, umbilicus partly opened; penial caecum short, internally with elongated verge formed by two main pilasters.

###### Description.

***Shell.*** Measurements (n = 11): SH 12.1–13.9 mm, SW 18.5–20.7 mm, AH 6.9–8.2 mm, AW 11.0–12.2 mm, W# 5.5–5.75, SH/SW 0.61–0.71; sinistral, with low conical spire, light brown to dark brown with red-brown peripheral band and umbilicus spot. Apex obtuse. Whorls regularly increasing, slightly convex. Periphery angulated. Base of shell convex. Surface completely covered with dense, fine, erected, curved, triangular, easily-dislodged scales and leaving crescent-shaped trace; upper surface with coarse, oblique axial ridges; spiral striation absent. Aperture roundly lunate. Peristome expanded; outer lip smoothly curved; columellar lip oblique, curve, joining curved basal lip smoothly or in an angle. Parietal callus smooth, thin, transparent. Umbilicus open, 2.6–3.2 mm in width, 1/5 covered by reflected columellar lip.

**Figure 2.**
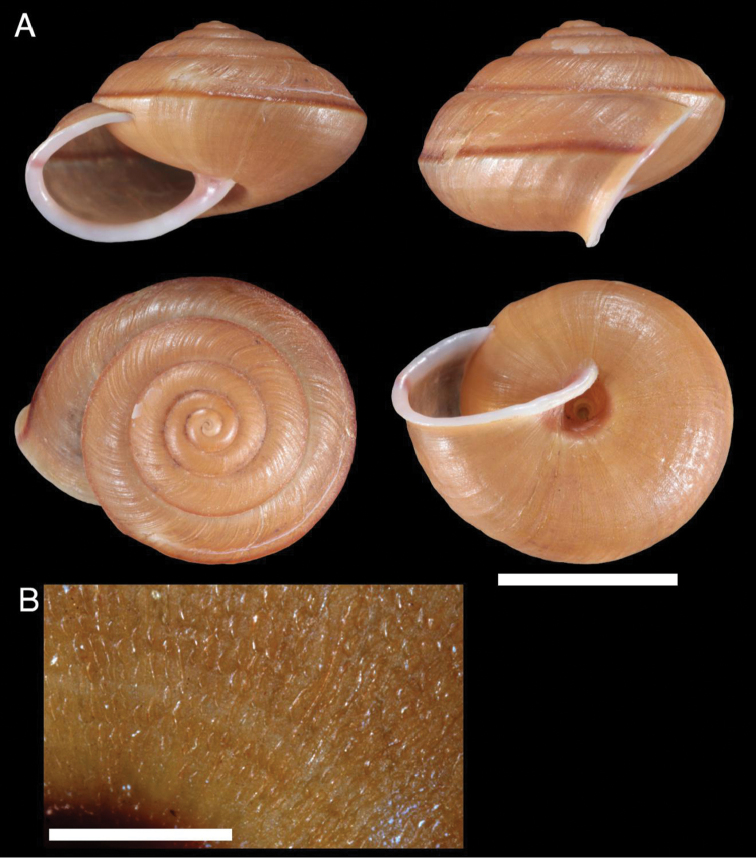
Shell of *Satsumasquamigera* sp. n. **A** shell of holotype (NMNS-7944-001) **B** scales on base of shell (paratype NMNS-794-002). Scale bar: 10 mm (**A**), 2 mm (**B**).

**Figure 3.**
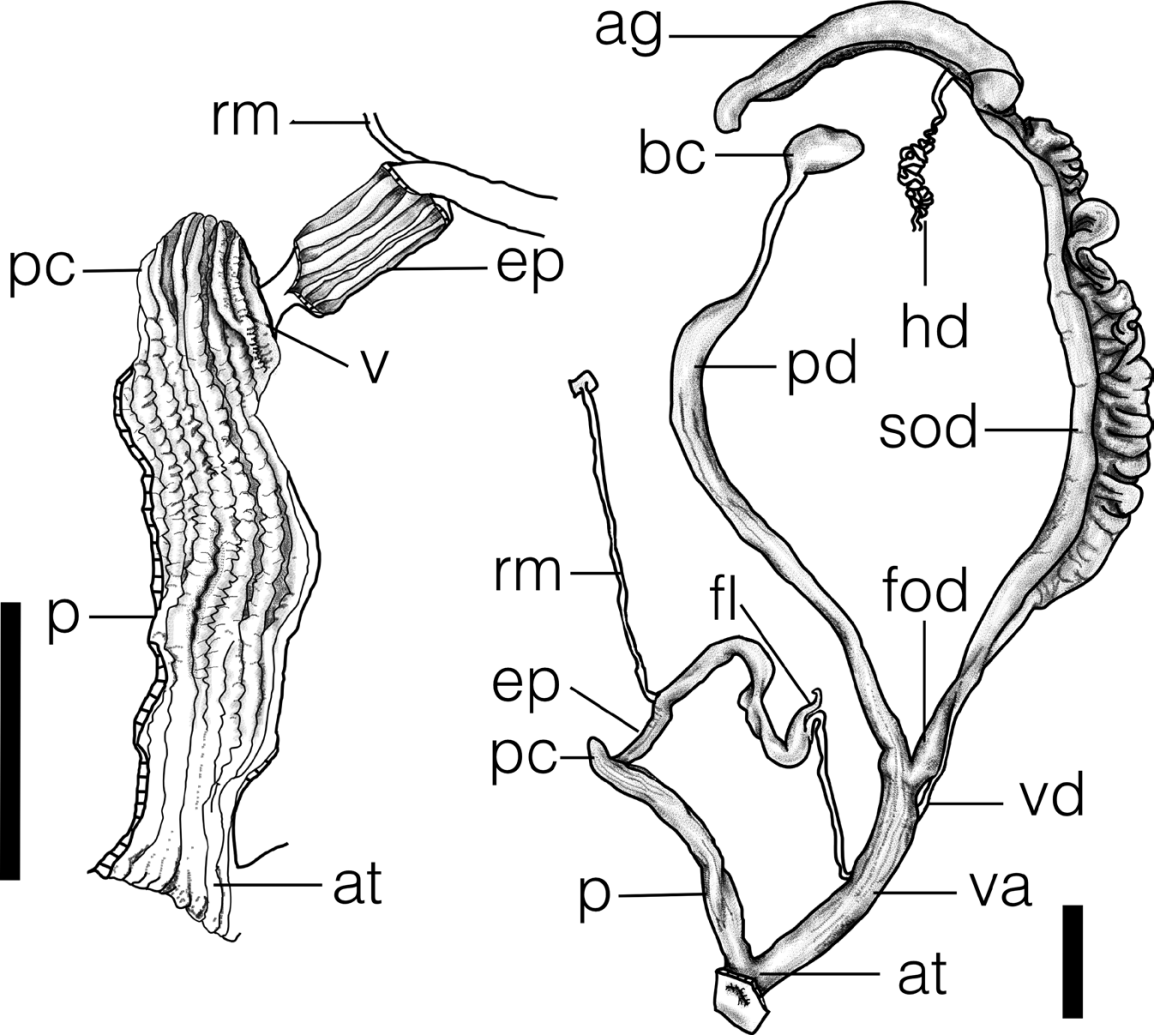
Reproductive system showing whole genitalia and opened penis of *Satsumasquamigera* sp. n. (holotype NMNS-7944-001). Scale bar: 5 mm.

***External morphology.*** Light brown with irregular, small, dark brown spots and a distinct yellowish line running from head between tentacles to collar. Tentacles dark brown.

***Reproductive system.*** Bursa copulatrix oval with long pedunculus of 27–30 mm. Free oviduct short. Vagina muscular, furrowed externally corresponding to internal folds, 10–12 mm in length. Atrium short, finely wrinkled inside. Penis slender, 10–12 mm in length, evenly thickened, furrowed externally corresponding to 7–8 strong, straight, corrugated pilasters internally. Penial caecum short, protruding 2–3 mm. Verge extending along penial caecum, formed by two main pilasters, with wrinkled surface. Epiphallus slender, 15–17 mm in length, internally with 4 smooth pilasters. Penis retractor muscle attached at distal 1/4 of epiphallus. Flagellum short, tapering.

###### Etymology.

From *squamigera* (Latin, adjective in the nominative feminine singular case) meaning scale-bearing, for the scaly shell surface.

###### Distribution.

This species was found in southern Pingtung County, including the type locality, Da-han-shan forest road (22°24.20'N; 120°45.31'E, alt-1555 m), Mt. Bei-li-long (22°11.81'N; 120°43.63'E, alt-320 m) and Mu-dang (22°8.43'N; 120°48.34'E, alt-240 m) (Figure 1A–D).

###### Ecology.

All specimens were collected in mountainous, lowland, broad-leafed forest. Mature adults were collected in mid-May and February, from ground, rocks or fallen tree trunks. This species is sympatric with the congeners *Satsumabacca* (Pfeiffer, 1866), *Satsumabatanicapancala* (Schmacker & Boettger, 1891) and *Satsumalongkiauwensis* Wu, Lin & Hwang, 2007.

###### Remarks.

*Satsumasquamigera* sp. n. is distinguished from all other sinistral species by having dense and curved scales on the whole shell surface. When fully matured, the scales typically fall off, leaving crescent-shaped granules. Some intact scales may remain beside sutures, on the base of the last whorl or inside the umbilicus. The new species is similar to *S.pekanensis* (Rolle, 1911) and *S.submeridionalis* (Zilch, 1951) in shape of shell and angulated periphery. In comparison to *S.pekanensis*, the new species has a shortened spire and an extended flagellum (Chang 1989). The new species differs from *S.submeridionalis* in having a slender base of pedunculus of bursa copulatrix and a regularly thickened proximal vagina (Wang et al. 2014).

##### Satsuma adiriensis sp. n.

Taxon classificationAnimaliaStylommatophoraCamaenidae

http://zoobank.org/676C67FF-BC84-4DE6-9D6E-12C30768DBB9

Figures 4 , 5

###### Type material.

Holotype NMNS-7945-001, dry shell, coll. C. C. Hwang, 24 Aug 1998, collected from type locality; paratype NMNS-7945-002, 1 specimen: dry shell and dissected soft part in ethanol, coll. S. C. Chang , 4 Jul 1997, Shan-ping, Liu-guei, Kaohsiung, 22°57.93'N; 120°41.28'E, alt. 850 m; paratype NMNS-7945-003, 1 dry shell, coll. C. C. Hwang, 25 May 1998, Mt. Fan-bao-jian, Nan-xi, Tainan, 22°71.48'N; 120°34.4'E, alt. 1000 m; paratype NMNS-7945-004, 1 dry subadult shell, coll. G. S. Hsiang, 29 Jun 1997, Ma-jia, Pingtung, 22°40.07'N; 120°40.65'E, alt. 1200 m.

###### Type locality.

Taiwan: Pingtung County, Wu-tai, A-li, 22°43.42'N; 120°45.44'E, alt. 1350 m, disturbed primary broad-leaf forest.

###### Diagnosis.

Shell sinistral, thin, fragile, smooth, with spaced, coarse ridges; periphery round, color band absent; umbilicus completely opened; penial caecum long, internally with elongated verge formed by two main pilasters.

###### Description.

***Shell.*** Measurements (n = 3): SH 10.8–12.9 mm, SW 17.4–20.6 mm, AH 6.9–8.5 mm, AW 8.6–10.3 mm, W# 5.5, SH/SW 0.61–0.68; sinistral, thin, fragile, semi-translucent, with low conic spire, light brown, without color band. Apex obtuse. Whorls regularly increasing, slightly convex. Periphery bluntly angulated on the first 3/4 of last whorl, becoming rounded 1/4 whorl before peristome. Base of shell convex. Surface covered with loose, coarse, oblique axial ridges, becoming thin on base; spiral striation absent. Aperture roundly lunate. Peristome expanded; outer lip smoothly curved; columellar lip sub-vertical, not reflected, joining with basal lip in a weak angle. Parietal callus smooth, thin, transparent. Umbilicus completely opened, 3.3–3.6 mm in width.

***External morphology.*** Light brown with dense, irregular, dark brown to black spots and a distinct yellowish line running from head between tentacles to collar. Tentacles dark brown.

***Reproductive system.*** Bursa copulatrix oval; pedunculus long, 31 mm in length, with slightly expanded base. Free oviduct short. Vagina thickened, smooth externally, with eleven internal pilasters, 11 mm in length. Atrium obvious, finely wrinkled inside. Penis muscular, 13 mm in length, evenly thickened, furrowed externally; distal half internally supporting three main, finely wrinkled pilasters; proximal half supporting eleven strong, corrugated pilasters. Penial caecum thickened, with blunt apex, protruding 7 mm. Verge extending along penial caecum, formed by two main pilasters. Epiphallus slender, 16 mm in length, internally with three smooth pilasters. Penis retractor muscle attached at distal 1/6 of epiphallus. Flagellum long, tapering, slightly wavy at middle portion.

**Figure 4.**
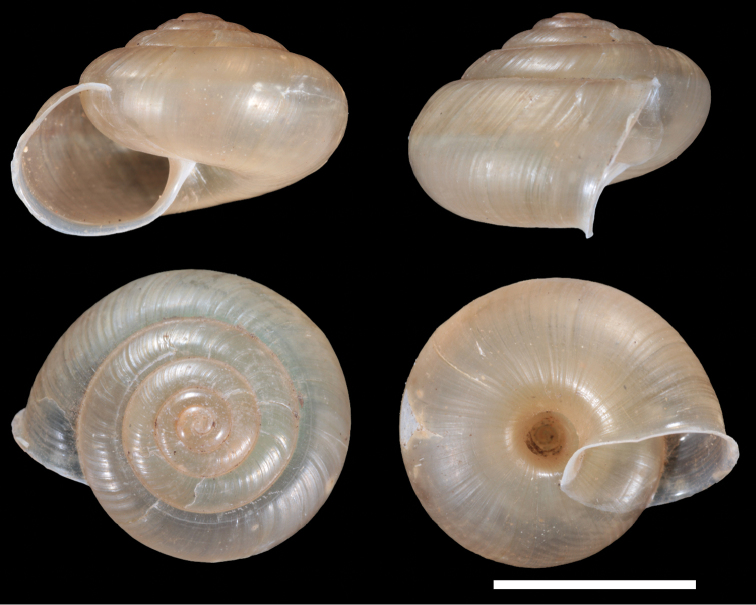
Shell of *Satsumaadiriensis* sp. n. (holotype NMNS-7945-001). Scale bar: 10 mm.

**Figure 5.**
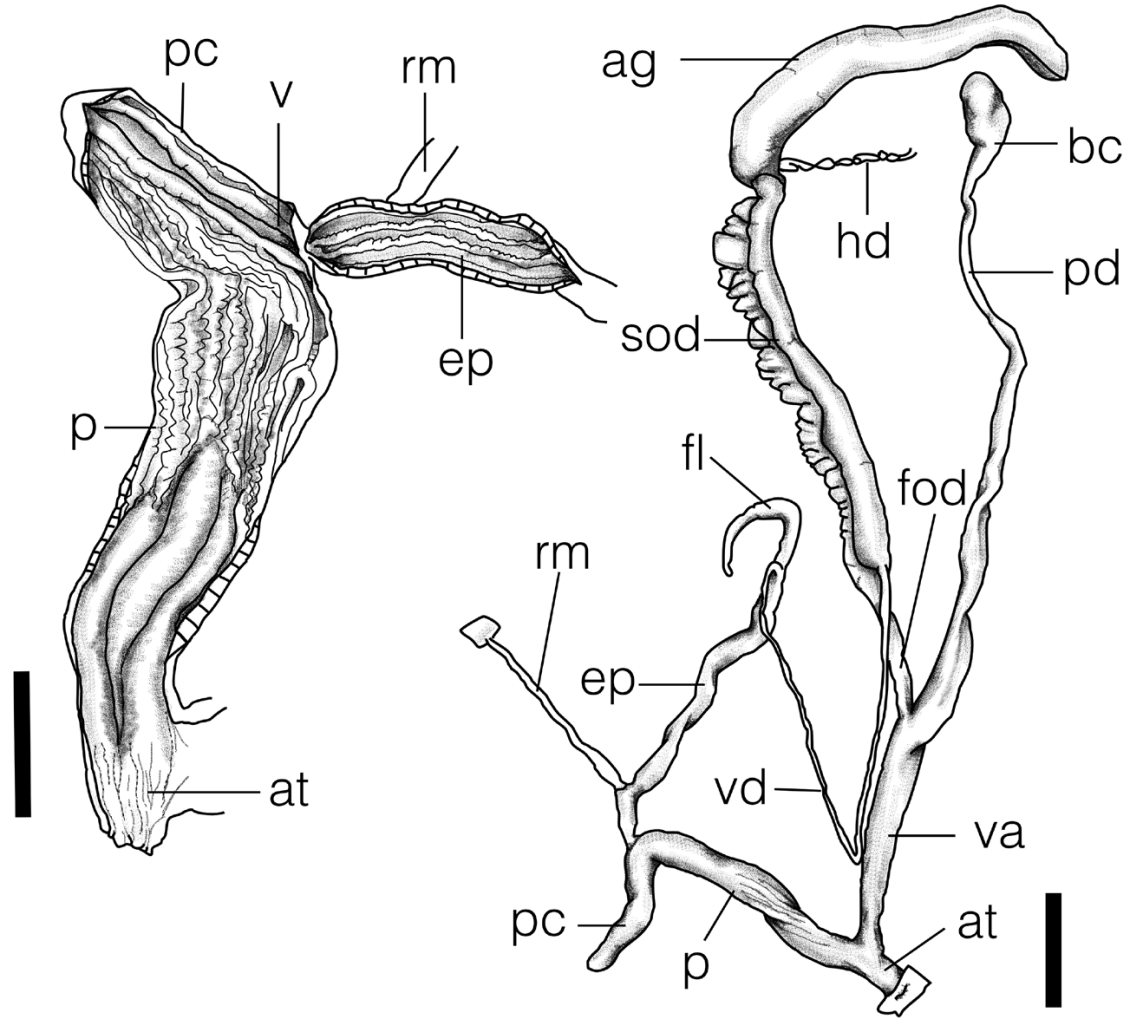
Reproductive system showing whole genitalia and opened penis of *Satsumaadiriensis* sp. n. (paratype NMNS-7945-002). Scale bar: 5 mm.

###### Etymology.

For Adiri, the indigenous Rukai name of the type locality, adjective of feminine gender.

###### Distribution.

Known from mid-elevation forest of Kaohsiung, Tainan and Pingtung (Figure 1E–H).

###### Ecology.

All specimens were collected in mountainous, mid-elevation, broad-leaf forest. The single live adult was collected in July, from a tree trunk. This species is sympatric with congeneric species *S.albida* (Adams, 1870) and *S.friesiana* (Moellendorff, 1884) at Shan-ping, *S.amblytropis* (Pilsbry, 1901) at Mt. Fan-bao-jian and an unknown *Satsuma*at the type locality A-li. Despite wide distribution in the mountainous areas of southwestern Taiwan, this species is quite rare.

###### Remarks.

*Satsumaadiriensis* sp. n. is similar to *S.contraria* (Pilsbry & Hirase, 1909), distributed in Kenting, Pingtung, in having a sinistral, semi-transparent shell with completely open umbilicus. The new species, however, has smaller shell width, round periphery on the final 1/4 of the last whorl, a sub-vertical columellar lip, a sinuous upper lip, coarse ridges on the surface, a slender pedunculus of bursa copulatrix, and a longer penial caecum and flagellum and shorter penis than the latter species (Hwang and Ger 2018).

The new species shares a sinistral and depressed conic shell with *Satsumaformosensis* (Pfeiffer, 1866) and *S.yaeyamensis* (Pilsbry, 1894), which are found in northern Taiwan and the Ryukyu Islands. *Satsumaadiriensis* differs from these two species by its thin, semi-transparent shell with loose, coarse surface ridges, a sub-vertical columellar lip joining basal lip in a weak angle, and a bluntly angulated periphery on the first 3/4 of the last whorl.

## Discussion

In this study, two new species of sinistral *Satsuma* were described based on shell and reproductive system characteristics. This work has brought the number of known sinistral *Satsuma* species to seventeen. Among these seventeen species, eleven are distributed in Taiwan, three in the Ryukyu Islands, two in southern China, and one in Batan Island, Philippines. The diversification of *Satsuma* has been explained by allopatric speciation (Kameda et al. 2007), prey-predator coevolution and chirality (Hoso et al. 2010), and arboreal behavior (Wu et al. 2008).

Periostracal ornamentations such as granules and hairs are commonly seen in confamilial genera, e.g., *Chloritis* Beck, 1837, *Moellendorffia* Ancey, 1887, *Aegista* Albers, 1850 and many genera from Australia (Solem 1984, Hirano et al. 2014, Criscione and Köhler 2016). In the genus *Satsuma*, granules on embryonic whorls are commonly seen (personal observations), but rarely reported. This under-reporting may be due to the ease with which these granules wear off, or their simply being so small as to evade observation. Three sinistral species, *S.perversa* (Pilsbry, 1931), *S.yaeyamensis* and *S.batanicapancala* have been observed to have granulate embryonic whorls (Azuma 1995, personal observations), however these species do not have scales covering the whole shell surface, as does *S.squamigera* sp. n.

Short, hooked hairs have been observed over the entire shell surface of the sinistral species *S.uncopila* (Heude, 1882). Granules on the entire shell surface are also reported in some dextral species, e.g., *S.ferruginea* (Pilsbry, 1900), *S.textilis* (Pilsbry & Hirase, 1904), *S.japonicagranulosa* (Pilsbry, 1902), *S.j.heteroglypta* (Pilsbry, 1900), *S.okiensis* (Pilsbry & Hirase, 1908) and *S.cristata* (Pilsbry, 1902). The hairs are thought to promote the snails’ adherence to leaves when humidity levels are high (Pfenninger et al. 2005). The evolutionary significance of these varying ornamentations of size, shape, and position remains questionable. This question will not be adequately answered until more complete phylogeny and comparative studies of the *Satsuma* genus become available.

**Author contributions**

CC Hwang performed the anatomical studies, executed this study, and wrote the manuscript; SP Wu helped with the data collecting and paper writing.

## Supplementary Material

XML Treatment for Satsuma squamigera

XML Treatment for Satsuma adiriensis

## References

[B1] Adams A Reeve L (1850) Mollusca, Part III. In: Adams A (Ed.) The Zoology of the Voyage of H.M.S. Samarang; Under the Command of Captain Sir Edward Belcher C.B., F.R.A.S., F.G.S., During the Years 1843–1846. Reeve, Benham & Reeve, London, 1–87.

[B2] Azuma M (1995) Colored Illustrations of the Land Snails of Japan. Hoikusha, 343 pp.

[B3] Chang KM (1989) Anatomy of *Coniglobusnuxpaiwanis* (Kuroda) and *Coniglobuspekanensis* (Rolle) from south Taiwan (Pulmonata: Camaenidae). Bulletin of Malacology 14: 1–8.

[B4] Criscione F Köhler F (2014) Molecular phylogenetics and comparative anatomy of *Kimberleytrachia* Köhler, 2011 – a genus of land snail endemic to the coastal Kimberley, Western Australia with description of new taxa (Gastropoda, Camaenidae). Contributions to Zoology 83: 245–267. http://www.ctoz.nl/vol83/nr04/a03

[B5] Criscione F Köhler F (2016) *Setobaudinianicolasi* – a new species from Baudin Island, Kimberley, Western Australia (Stylommatophora, Camaenidae). Molluscan Research 36: 290–293. 10.1080/13235818.2016.1201037

[B6] Gómez BJ (2001) Structure and functioning of the reproductive system. In: Baker GM (Ed.) The Biology of Terrestrial Molluscs.CABI Publishing, Oxon, 307–330. 10.1079/9780851993188.0307

[B7] Hirano T Kameda Y Kimura K Chiba S (2014) Substantial incongruence among the morphology, taxonomy, and molecular phylogeny of the land snails *Aegista*, *Landouria*, *Trishoplita*, and *Pseudobuliminus* (Pulmonata: Bradybaenidae) occurring in East Asia. Molecular Phylogenetics and Evolution 70: 171–181. 10.1016/j.ympev.2013.09.020 24096054

[B8] Hoso M Kameda Y Wu SP Asami T Kato M Hori M (2010) A speciation gene for left-right reversal in snails results in anti-predator adaptation. Nature Communications 1: 133. 10.1038/ncomms1133 PMC3105295 21139578

[B9] Hsieh BC Wu SP Tsai CL (2013) Land Snails of Taiwan (3^rd^ edn). Forestry Bureau, Council of Agriculture, Executive Yuan, Taiwan, 381 pp.

[B10] Hwang CC Ger MJ (2018) Reproductive system of land snail *Satsumacontraria* (Stylommatophora: Camaenidae). Bulletin of Malacology 41: 36–45.

[B11] Hwang CC Okubo K Tada A (2017) *Satsumajinlunensis* – a new species from Taiwan (Stylommatophora: Camaenidae). Molluscan Research. 10.1080/13235818.2017.1358340

[B12] Kameda Y Kawakita A Kato M (2007) Cryptic genetic divergence and associated morphological differentiation in the arboreal land snail Satsuma (Luchuhadra) largillierti (Camaenidae) endemic to the Ryukyu Archipelago, Japan. Molecular Phylogenetics and Evolution 45: 519–533. 10.1016/j.ympev.2007.03.021 17500012

[B13] Kerney MP Cameron RAD (1979) Land Snails of Britain & North-west Europe. Harper & Collins, London, 288 pp.

[B14] Kuroda T Habe T (1949) Helicacea. Sanmeisha, Tokyo, 129 pp.

[B15] Minato H (1988) A Systematic and Bibliographic List of the Japanese Land Snails. Shirahama, Japan, 294 pp.

[B16] Pfenninger M Hrabáková M Steinke D Dèpraz A (2005) Why do snails have hairs? A Bayesian inference of character evolution. BMC Evolutionary Biology 5: 59. 10.1186/1471-2148-5-59 PMC1310604 16271138

[B17] QGIS Development Team (2016) QGIS Geographic Information System. Open Source Geospatial Foundation Project. http://www.qgis.org

[B18] Schileyko AA (2004) Treatise on Recent terrestrial pulmonate molluscs, Part 12: Bradybaenidae, Monadeniidae, Xanthonychidae, Epiphragmophoridae, Helminthoglyptidae, Elonidae, Humboldtianidae, Sphincterochilidae, Cochlicellidae. Ruthenica, supplement 2: 1627–1763.

[B19] Schileyko AA (2011) Check-list of land pulmonate molluscs of Vietnam (Gastropoda: Stylommatophora). Ruthenica 21: 1–68. https://biotaxa.org/Ruthenica/article/view/3603

[B20] Schneider CA Rasband WS Eliceiri KW (2012) NIH Image to ImageJ: 25 years of image analysis. Nature Methods 9: 671–675. 10.1038/nmeth.2089 22930834 PMC5554542

[B21] Solem A (1984) Camaenid land snails from Western and central Australia (Mollusca: Pulmonata: Camaenidae). IV. Taxa from the Kimberley, Westraltrachia Iredale, 1933 and related genera. Records of the Western Australian Museum, Supplement 17: 427–705.

[B22] Wade CM Hudelot C Davison A Naggs F Mordan PB (2007) Molecular phylogeny of the helicoid land snails (Pulmonata: Stylommatophora: Helicoidea), with special emphasis on the Camaenidae. Journal of Molluscan Studies 73: 411–415. 10.1093/mollus/eym030

[B23] Wang P Xiao Q Zhou WC Hwang CC (2014) Revision of three camaenid and one bradybaenid species (Gastropoda, Stylommatophora) from China based on morphological and molecular data, with description of a new bradybaenid subspecies from Inner Mongolia, China. ZooKeys 372: 1–16. 10.3897/zookeys.372.6581 PMC3909801 24493955

[B24] Wu SP Hwang CC Lin YS (2008) Systematic revision of the arboreal snail *Satsumaalbida* species complex (Mollusca: Camaenidae) with descriptions of 14 new species from Taiwan. Zoological Journal of the Linnean Society 154: 437–493. 10.1111/j.1096-3642.2008.00415.x

[B25] Wu SP Lin YS Hwang CC (2007) A new *Satsuma* species (Pulmonata: Camaenidae) endemic to Taiwan. Zootaxa 1608: 59–68.

[B26] Wu SP Tsai CL (2014) A new sinistral *Satsuma* land snail (Pulmonata: Camaenidae) endemic to Taiwan. Bulletin of Malacology 37: 61–72.

[B27] Wu SP Tsai CL (2015) A new endemic dextral *Satsuma* land snail (Pulmonata: Camaenidae) from Taiwan. Bulletin of Malacology 38: 41–48.

[B28] Wu SP Tsai CL (2016) A new dextral species land snail of genus *Satsuma* (Pulmonata: Camaenidae) endemic to Taiwan. Bulletin of Malacology 39: 47–58.

[B29] Wu SP Wu CC (2017a) A new and endemic sinistral *Satsuma* land snail (Pulmonata: Camaenidae) from South Taiwan. Bulletin of Malacology 40: 27–42.

[B30] Wu SP Wu CC (2017b) A new dextral land snail of genus *Satsuma* (Pulmonata: Camaenidae) endemic to Taiwan. Bulletin of Malacology 40: 13–26.

